# Placental Transcriptome Adaptations to Maternal Nutrient Restriction in Sheep

**DOI:** 10.3390/ijms22147654

**Published:** 2021-07-17

**Authors:** Chelsie B. Steinhauser, Colleen A. Lambo, Katharine Askelson, Gregory W. Burns, Susanta K. Behura, Thomas E. Spencer, Fuller W. Bazer, Michael Carey Satterfield

**Affiliations:** 1 Department of Animal Science, Texas A & M University, College Station, TX 77843, USA; csteinhauser@tamu.edu (C.B.S.); kbeaso@gmail.com (K.A.); fbazer@tamu.edu (F.W.B.); 2 Department of Veterinary Physiology and Pharmacology, Texas A & M University, College Station, TX 77843, USA; clambo@tamu.edu; 3 Department of Obstetrics, Gynecology and Reproductive Biology, Michigan State University, Grand Rapids, MI 49503, USA; burnsgr2@msu.edu; 4 Division of Animal Sciences, University of Missouri, Columbia, MO 65211, USA; behuras@missouri.edu (S.K.B.); spencerte@missouri.edu (T.E.S.); 5 Institute for Data Science and Informatics, University of Missouri, Columbia, MO 65211, USA

**Keywords:** placentome, pregnancy, nutrient restriction, gene expression

## Abstract

Placental development is modified in response to maternal nutrient restriction (NR), resulting in a spectrum of fetal growth rates. Pregnant sheep carrying singleton fetuses and fed either 100% (*n* = 8) or 50% (NR; *n* = 28) of their National Research Council (NRC) recommended intake from days 35–135 of pregnancy were used to elucidate placentome transcriptome alterations at both day 70 and day 135. NR fetuses were further designated into upper (NR NonSGA; *n* = 7) and lower quartiles (NR SGA; *n* = 7) based on day 135 fetal weight. At day 70 of pregnancy, there were 22 genes dysregulated between NR SGA and 100% NRC placentomes, 27 genes between NR NonSGA and 100% NRC placentomes, and 22 genes between NR SGA and NR NonSGA placentomes. These genes mediated molecular functions such as MHC class II protein binding, signaling receptor binding, and cytokine activity. Gene set enrichment analysis (GSEA) revealed significant overrepresentation of genes for natural-killer-cell-mediated cytotoxicity in NR SGA compared to 100% NRC placentomes, and alterations in nutrient utilization pathways between NR SGA and NR NonSGA placentomes at day 70. Results identify novel factors associated with impaired function in SGA placentomes and potential for placentomes from NR NonSGA pregnancies to adapt to nutritional hardship.

## 1. Introduction

In eutherian mammals, the placenta mediates the exchange of nutrients, gases, and waste products between mother and fetus. Impaired growth and function of the placenta is associated with fetal growth restriction, poor pregnancy outcomes, and susceptibility to a myriad of health-related consequences in adulthood [1,2,3,4]. Placental growth can be influenced by maternal exposure to environmental factors, such as malnutrition. An area of increasing interest is the elucidation of adaptive mechanisms by which the placenta can respond to maternal environmental insults in a compensatory manner to sustain adequate fetal growth despite, for example, maternal nutrient restriction.

Using a long-term nutritionally restricted pregnant sheep model, we recently reported that nutrient-restricted (NR) pregnant sheep that support rates of fetal growth similar to growth of fetuses in control-fed sheep exhibited increased expression of select amino acid transporters in the placenta and possessed increased amino acid availability in the fetal circulation [5]. In the same cohort of animals, microarray analyses revealed changes in the expression of genes whose functions were associated with the biological actions of nutrient sensing and transport and immune system activation [6]. While the aforementioned sheep studies highlight adaptive changes occurring within the ovine placenta to support normal fetal growth during maternal NR, they are limited in that analyses were performed during the final third of gestation, when placental growth and function had already reached its maximum [7]. This in fact highlights one of the biggest challenges of placental research: how do we assess early placental growth and function, while allowing pregnancy to progress, giving the opportunity to definitively link early placental growth, or early placental adaptations, with a late gestation fetal phenotype? The sheep serves as a unique and valuable model organism to address this dilemma.

The primary functional units of the sheep placenta are the placentomes, which are discrete regions where the maternal caruncle intimately interdigitates and syncytializes with the fetal cotyledon. A singleton pregnancy contains between 50 and 120 of these placentomes, which collectively support greater than 95% of the hematotrophic exchange between mother and fetus [8]. We recently developed a surgical technique to selectively remove a single placentome in close approximation to the fetus in mid-gestation without compromising fetal growth [9]. Using this approach, we have identified alterations in placental fatty acid transport in NR pregnancies with impaired fetal growth that correspond to changes in circulating levels of triglycerides, non-esterified fatty acids, and cholesterol in both the dam and the fetus [10]. Additionally, thyroid hormones are altered in NR pregnancies, with identifiable changes in placental thyroid-hormone-related genes and proteins during mid and late gestation [11].

Given the demonstrated potential of our surgical technique, the objective of the present study was to utilize a discovery-based approach to identify novel genes and biological processes associated with the earliest adaptations within the ovine placentome in response to maternal NR, due to total caloric restriction from day 35 to day 135 (term = day 147) of pregnancy, giving rise to either small-for-gestational-age (SGA) or normal-weight (NonSGA) fetuses in late gestation.

## 2. Results

### 2.1. Model Characteristics

Maternal, placental, and fetal weights, as well as select metabolite abundances for this study, have been published elsewhere [10,11,12,13]. Of importance, fetuses from NR dams were categorized by fetal weight at day 135 into quartiles, with the highest quartile being denoted as NR NonSGA (*n* = 7) and the lowest quartile as NR SGA (*n* = 7). Well-fed controls are denoted as 100% NRC (*n* = 8). Fetal weight was lower in the NR SGA (3.8 ± 0.2 kg) compared to 100% NRC (5.6 ± 0.1 kg) and NR NonSGA (5.4 ± 0.2 kg; *p* < 0.001) fetuses [12]. Total placentome weight was lower in NR SGA (307 ± 16 g) ewes compared to 100% NRC (546 ± 43 g) and NR NonSGA (524 ± 36 g; *p* < 0.001) pregnancies [10].

### 2.2. Differentially Expressed Genes

**Figure 1:**
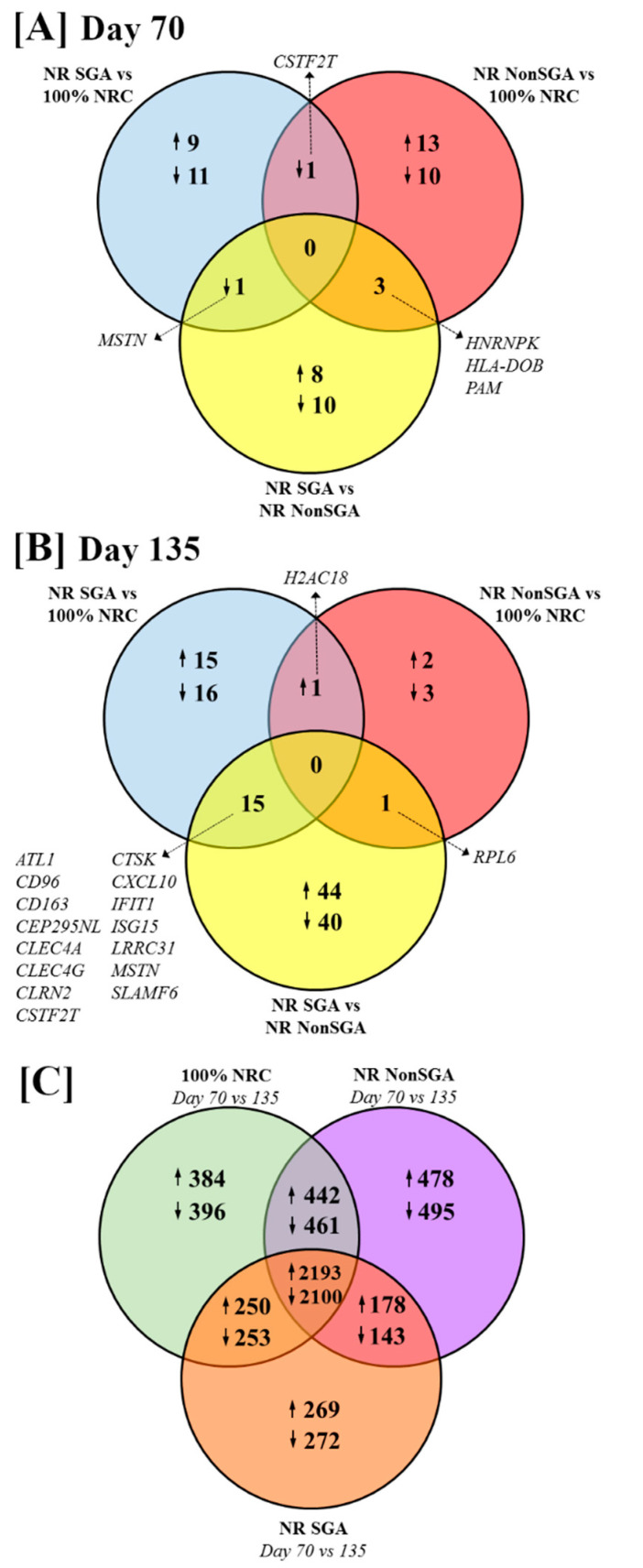
Venn diagrams depicting differentially expressed genes (DEGs) between 100% NRC (*n* = 8), NR NonSGA (*n* = 7), and NR SGA (*n* = 7) placentomes at day 70 (**A**) and day 135 (**B**) of gestation. The number of DEGs between day 70 and day 135 within each treatment group is depicted in (**C**). Genes were considered differentially expressed when FDR ≤ 0.10 and *p* ≤ 0.05.

The numbers of differentially expressed genes (DEGs) between treatment groups and days of pregnancy are shown in Figure 1 (FDR ≤ 0.10 and *p* ≤ 0.05). At day 70 of pregnancy, 9 genes were upregulated and 11 downregulated in NR SGA compared to 100% NRC placentomes, while 13 genes were upregulated and 10 genes downregulated in NR NonSGA compared to 100% NRC placentomes (Figure 1A). *Cleavage stimulation factor subunit 2 tau* (*CSTF2T*) was downregulated in both NR SGA and NR NonSGA placentomes compared to 100% NRC placentomes. There were 8 genes upregulated and 10 genes downregulated in NR SGA compared to NR NonSGA placentomes (Figure 1A). *Myostatin* (*MSTN*) was downregulated in NR SGA compared to both 100% NRC and NR NonSGA placentomes (Figure 1A, Table 1). *Heterogeneous nuclear ribonucleoprotein K* (*HNRNPK*) was upregulated in 100% NRC, but downregulated in NR SGA placentomes compared to NR NonSGA placentomes. *Major histocompatibility complex, class II, DO beta* (*HLA-DOB*) and *peptidylglycine alpha-amidating monooxygenase* (*PAM*) were downregulated in 100% NRC placentomes compared to all NR placentomes (Figure 1A, Table 1).

At day 135 of pregnancy, 15 genes were upregulated and 16 genes downregulated in NR SGA compared to 100% NRC placentomes, while only 2 genes were upregulated and 3 genes downregulated in NR NonSGA compared to 100% NRC placentomes (Figure 1B). *H2A clustered histone 18* (*H2AC18*) was upregulated in all NR placentomes compared to 100% NRC placentomes (Figure 1B, Table 2). There were 44 genes upregulated and 40 downregulated in NR SGA compared to NR NonSGA placentomes (Figure 1B). *Ribosomal protein L6* (*RPL6*) was downregulated in 100% NRC and NR SGA compared to NR NonSGA placentomes (Figure 1B, Table 2). *CD96, CD163, C-type lectin domain family 4 member A* (*CLEC4A*)*, CLEC4G, CSTF2T, cathepsin K* (*CTSK*)*, C-X-C motif chemokine ligand 10* (*CXCL10*)*, interferon-induced protein with tetratricopeptide repeats 1* (*IFIT1*)*, interferon-stimulated gene 15* (*ISG15*)*, leucine-rich repeat-containing 31* (*LRRC31*)*,* and *signaling lymphocyte activation molecule family member 6* (*SLAMF6*) were upregulated in NR SGA compared to 100% NRC and NR NonSGA placentomes, while *glutamic pyruvic transaminase* (*ALT1*), *centrosomal protein 295 n-terminal like* (*CEP295NL*), *clarin-2* (*CLRN2*), and *MSTN* were downregulated in NR SGA placentomes at day 135 of pregnancy (Figure 1B, Table 2).

The majority of genes that are differently expressed between days 70 and 135 of pregnancy are the same between 100% NRC, NR NonSGA, and NR SGA placentomes (2193 genes upregulated, 2100 genes downregulated; Figure 1C). Interestingly, there are fewer genes overall that are differentially expressed between days in the NR SGA placentomes (5658 genes) compared to NR NonSGA (6490 genes) and 100% NRC (6479 genes) placentomes (Figure 1C). Additionally, 100% NRC and NonSGA placentomes have more differentially expressed genes in common (442 upregulated, 461 downregulated) than either group does with NR SGA placentomes (250 up- and 253 downregulated, and 178 up- and 143 downregulated, respectively; Figure 1C).

All DEGs identified by treatment comparison at day 70 of pregnancy are listed in Table 1, and those from day 135 are listed in Table 2.

### 2.3. Over-Representation Analysis of GO: Molecular Functions

**Figure 2:**
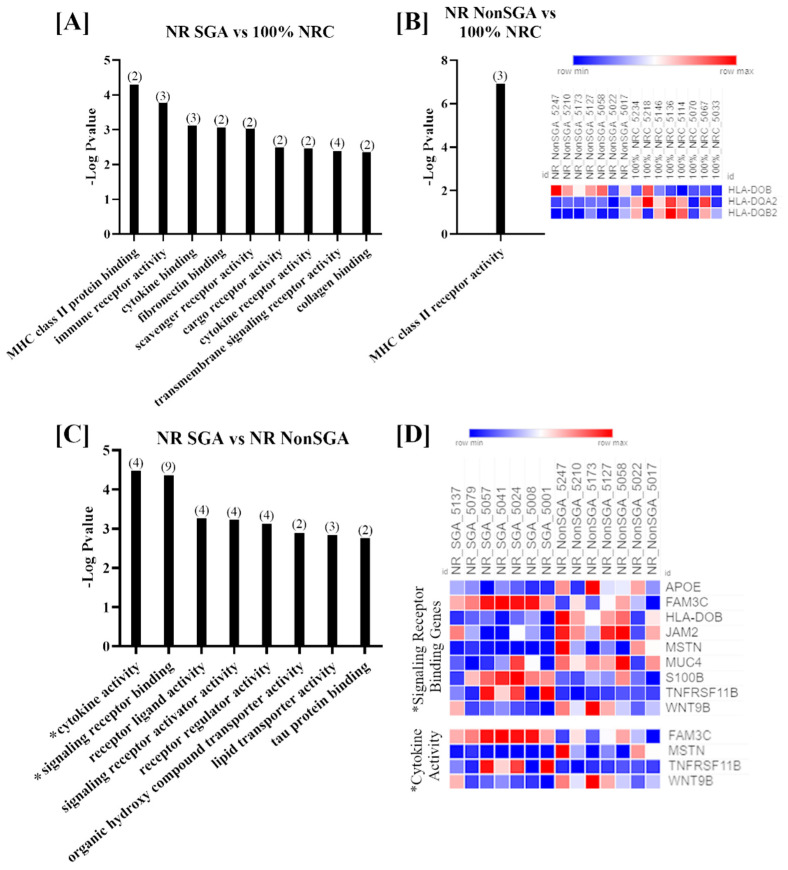
GO molecular function analyses of differentially expressed genes (DEGs) in placentomes at day 70 of pregnancy. Molecular functions differentially regulated in NR SGA compared to 100% NRC placentomes (**A**). The molecular function differentially regulated in NR NonSGA compared to 100% NRC placentomes, with a heat map of the three involved genes (**B**). Molecular functions differentially regulated in NR SGA compared to NR NonSGA placentomes (**C**), * with a heat map of genes involved in “signaling receptor binding” and “cytokine activity” (**D**). The heat maps correspond to one sample for each column and one gene for each row. All depicted GO molecular functions were significant at FDR < 0.05. Numbers in parentheses indicate the number of DEGs involved in the function.

Genes that were differentially expressed between the three treatment groups on day 70 of pregnancy were further analyzed to determine changes in Gene Ontology (GO) molecular functions (Figure 2). Only functions with > 1 gene involved and an FDR < 0.05 are shown. While the number of DEGs was fairly low in each comparison at day 70, there were still nine functions that were differentially enriched between NR SGA and 100% NRC placentomes, including terms such as MHC class II protein binding, cytokine binding, and transmembrane signaling receptor binding (Figure 2A). MHC class II receptor activity was the only function noted in NR NonSGA compared to 100% NRC placentomes, and the heat map shows that two of the three involved genes (*major histocompatibility complex, class II, DQ alpha 2* (*HLA-DQA2*) and *major histocompatibility complex, class II, DQ beta 2* (*HLA-DQB2*)) were downregulated in NR NonSGA compared to 100% NRC placentomes, while *HLA-DOB* was upregulated in NR NonSGA placentomes (Figure 2B). In NR SGA compared to NR NonSGA placentomes there were eight functions noted that included terms such as receptor ligand activity, organic hydroxyl compound transporter activity, and lipid transporter activity (Figure 2C). Signaling receptor binding and cytokine activity were also functions of note identified in NR SGA versus NR NonSGA placentomes, and as they were also identified at day 135, the expression for the genes involved in those functions is shown in a heat map in Figure 2D. Genes that are upregulated in NR SGA placentomes include *family with sequence similarity 3 member C* (*FAM3C*), *S100 calcium-binding protein B* (*S100B*), and *osteoprotegerin* (*TNFRSF11B*), while *apolipoprotein E* (*APOE*), *HLA-DOB*, *junctional adhesion molecule 2* (*JAM2*), *MSTN*, and *Wnt family member 9B* (*WNT9B*) were downregulated.

**Figure 3:**
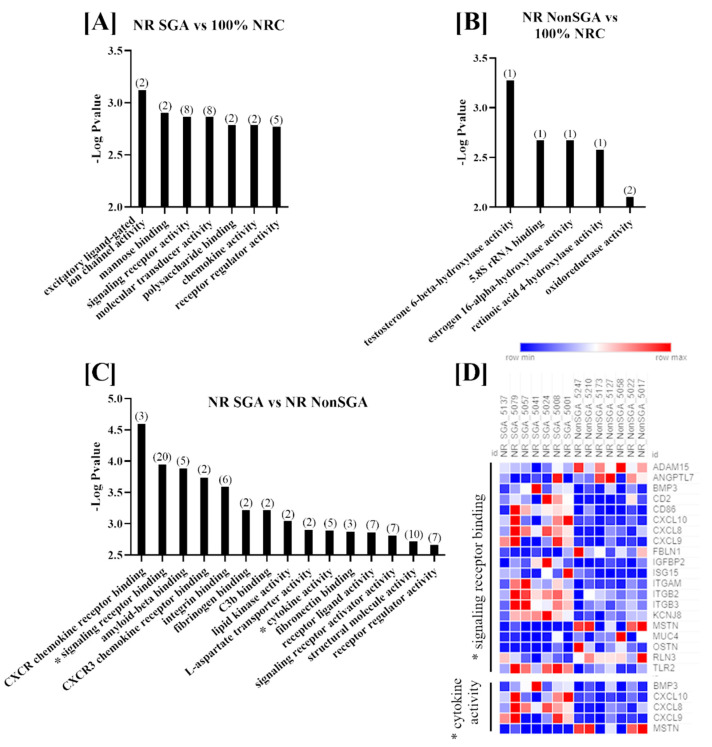
GO molecular function analyses of differentially expressed genes (DEGs) in placentomes at day 135 of pregnancy. Molecular functions differentially regulated in NR SGA compared to 100% NRC placentomes (**A**), NR NonSGA compared to 100% NRC placentomes (**B**), and NR SGA compared to NR NonSGA placentomes (**C**), * with a heat map of genes involved in “signaling receptor binding” and “cytokine activity” (**D**). The heat map corresponds to one sample for each column and one gene for each row. All depicted GO molecular functions were significant at FDR < 0.05. Numbers in parentheses indicate the number of differentially expressed genes involved in the function.

Molecular functions that were differentially enriched between treatment groups at day 135 are shown in Figure 3. There were seven functions altered between NR SGA and 100% NRC placentomes, which included terms such as signaling receptor binding, molecular transducer activity, and polysaccharide binding (Figure 2A). Since only seven genes were differentially expressed between NR NonSGA and 100% NRC placentomes, only functions involving one or two genes were identified; those functions include hydroxylase activity for multiple molecules and oxidoreductase activity (Figure 3B). There were 15 functions with >1 gene altered in NR SGA compared to NR NonSGA placentomes, with terms such as CXCR chemokine receptor binding, amyloid-beta binding, and lipid kinase activity (Figure 3C). As at day 70, signaling receptor binding and cytokine activity were two functions with significant differences between NR SGA and NR NonSGA placentomes. The genes *MSTN* and *MUC4* continued to be downregulated in day 135 NR SGA placentomes, but none of the other genes identified in these functions at day 70 were still differentially regulated at day 135. Interestingly, multiple members of the C–X–C motif chemokine family (*CXCL8*, *CXCL9*, *CXCL10*) were upregulated in the NR SGA placentomes, as were multiple integrins (*ITGAM*, *ITGB2*, *ITGB3*; Figure 3D).

### 2.4. Transcription Factors Potentially Regulating DEGs

**Figure 4:**
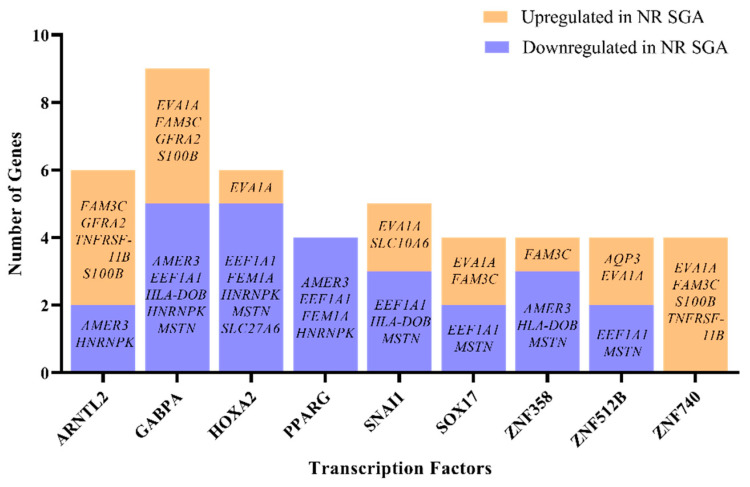
Computational analysis of transcription factors potentially regulating differentially expressed genes (DEGs) in NR SGA compared to NR NonSGA placentomes at day 70 of pregnancy. Only transcription factors that are expressed in placentomes and have ≥4 gene targets are depicted.

There is a single known transcription factor—*zinc finger protein 462* (*ZNF462*)—that is differentially expressed in NR SGA compared to NR NonSGA placentomes at day 70, but little is known about which genes are specifically regulated by *ZNF462*. Therefore, the list of differentially expressed genes between NR SGA and NR NonSGA placentomes at day 70 was analyzed to determine transcription factors that are expressed in day 70 placentomes that could potentially be regulating gene expression at that time. There were nine transcription factors (*aryl hydrocarbon receptor nuclear translocator like 2* (*ARNTL2*), *GA-binding protein transcription factor subunit alpha* (*GABPA*), *homeobox A2* (*HOXA2*), *peroxisome proliferator-activated receptor gamma* (*PPARG*), *snail family transcriptional repressor 1* (*SNAI1*), *SRY-box transcription factor 17* (*SOX17*), *ZNF358*, *ZNF512B*, *ZNF740*) identified that potentially have the motifs to regulate at least 4 of the 22 identified DEGs (Figure 4). Of specific interest, *GABPA* has motifs to regulate nine of the DEGs, while all four genes with *PPARG* motifs are downregulated in SGA placentomes and all four genes with *ZNF740* motifs are upregulated in SGA placentomes (Figure 4).

### 2.5. Gene Set Enrichment Analysis (GSEA) of Hallmark Pathways

Due to the relatively small number of DEGs between treatment groups, GSEA was used to further identify pathways that were specifically altered in NR SGA compared to NR NonSGA placentomes. Of note, sheep Ensembl IDs were converted to human Ensembl IDs before performing GSEA analyses due to limitations in the availability of sheep-related resources; thus, any sheep-specific genes were unable to be assessed as part of the GSEA analyses.

**Figure 5:**
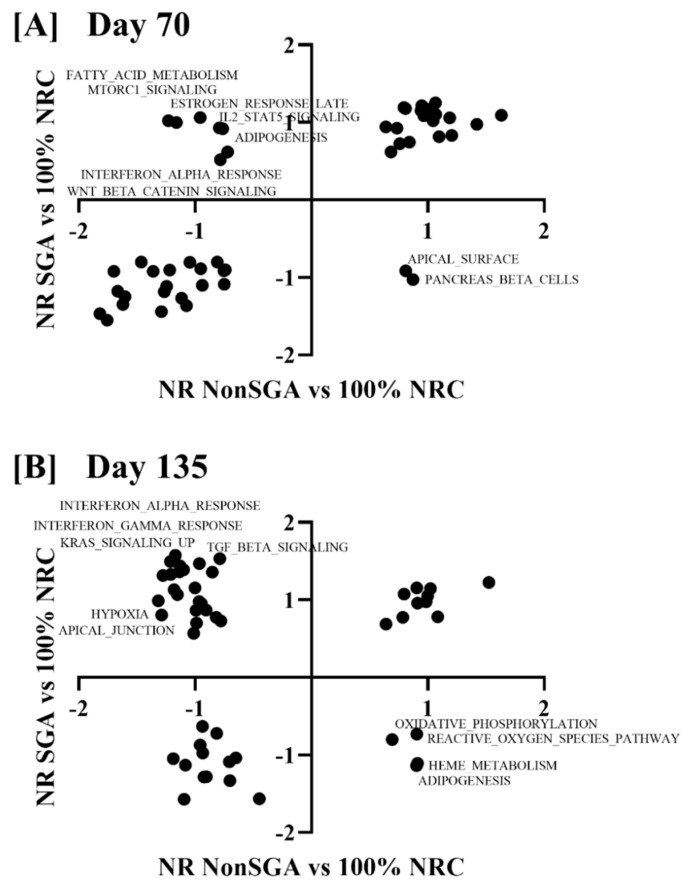
Gene set enrichment analysis of hallmark pathways in NR SGA compared to NR NonSGA placentomes at day 70 (**A**) and day 135 (**B**) of pregnancy. Nominal enrichment scores (NES) of NR NonSGA vs. 100% NRC analyses were plotted against NES values from NR SGA vs. 100% NRC analyses. Points in quadrants with (+, −) or (−, +) values were considered differentially regulated and were labeled with their pathway name.

Nominal enrichment score (NES) values for the 50 hallmark pathways from an NR NonSGA versus 100% NRC analysis were plotted against those from a NR SGA versus 100% NRC analysis, with any point falling into a (+, −) or (−, +) quadrant being considered different between NR SGA and NR NonSGA placentomes (Figure 5). At day 70, apical surface and pancreas beta cell pathways were upregulated in NR NonSGA, but not NR SGA placentomes (Figure 5A). Additionally, pathways including fatty acid metabolism, MTORC1 signaling, IL2-STAT5 signaling, and Wnt/β-catenin signaling were upregulated in NR SGA, but not NR NonSGA placentomes (Figure 5A). At day 135, oxidative phosphorylation, reactive oxygen species, heme metabolism, and adipogenesis pathways were upregulated in NR NonSGA, but not NR SGA pathways. Pathways such as interferon alpha/gamma response, KRAS signaling, TGF-β signaling, hypoxia, and apical junctions were upregulated in NR SGA, but not NR NonSGA placentomes.

**Figure 6:**
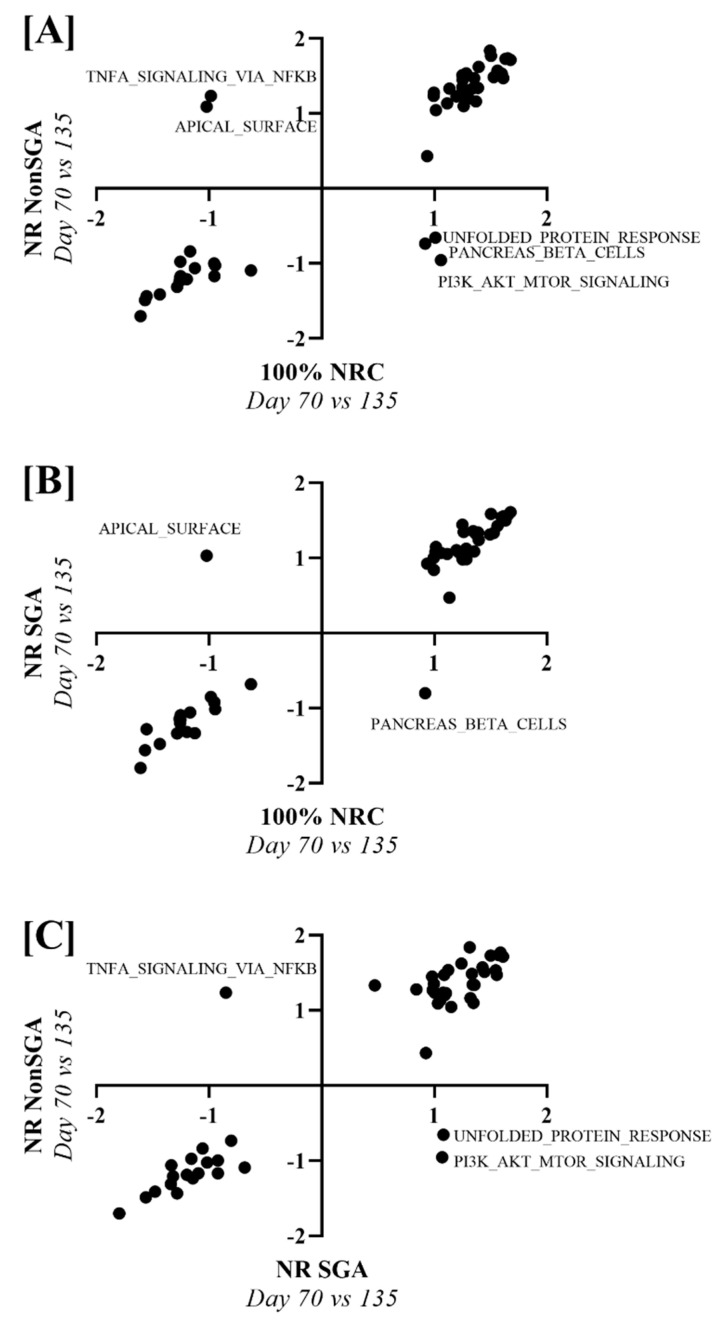
Gene set enrichment analysis of hallmark pathways between treatment groups across days of pregnancy. Nominal enrichment scores (NES) of NR NonSGA (day 70 vs. 135) analyses were plotted against NES values from 100% NRC (day 70 vs. 135) analyses (**A**). NES values of NR SGA (day 70 vs. 135) analyses were plotted against 100% NRC (day 70 vs. 135) analyses NES values (**B**). NES values of NR NonSGA (day 70 vs. 135) analyses were plotted against NR SGA (day 70 vs. 135) analyses NES values (**C**). Points in quadrants with (+, −) or (−, +) values were considered differentially regulated, and were labeled with their pathway name.

To determine pathways that changed differently over time between 100% NRC, NR NonSGA, and NR SGA placentomes, NES values from a day 70 versus day 135 analysis of each treatment group were plotted against one another (Figure 6). Unfolded protein response, pancreas beta cells, and PI3K–AKT–MTOR signaling pathways were upregulated in 100% NRC placentomes at day 70, but not NR NonSGA, while TNF-α signaling via NF-κB and apical surface pathways were upregulated in NR NonSGA, but not 100% NRC placentomes (Figure 6A). The pancreas beta cell pathway was also upregulated in 100% NRC placentomes at day 70, but not NR SGA placentomes, while the apical surface pathway was upregulated in NR SGA, but not 100% NRC placentomes (Figure 6B). Pathways upregulated in NR SGA, but not NR NonSGA, included unfolded protein response and PI3K–AKT–MTOR signaling, while TNF-α signaling via NF-κB was upregulated in NR NonSGA, but not NR SGA placentomes (Figure 6C).

### 2.6. Gene Set Enrichment Analysis (GSEA) of KEGG Pathways

**Figure 7:**
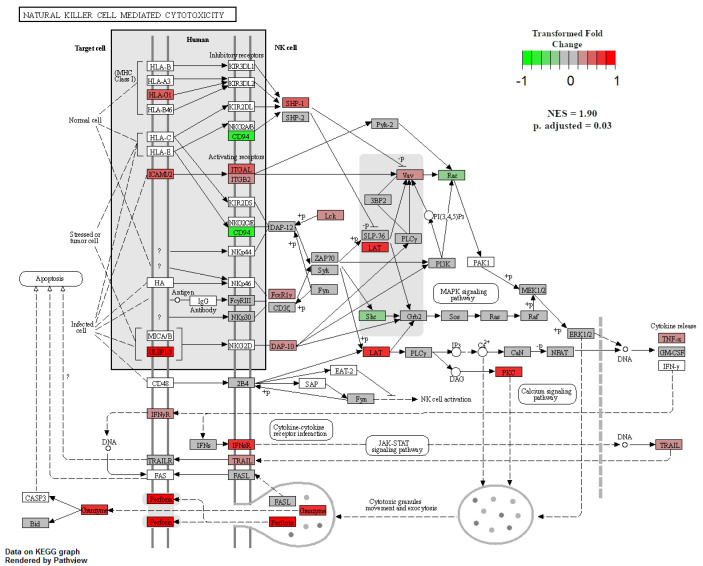
Natural-killer-cell-mediated cytotoxicity in NR SGA versus 100% NRC placentomes at day 70 of pregnancy. Gene set enrichment analysis was performed to identify KEGG pathways that were significantly enriched at adjusted *p*-values < 0.05.

**Figure 8:**
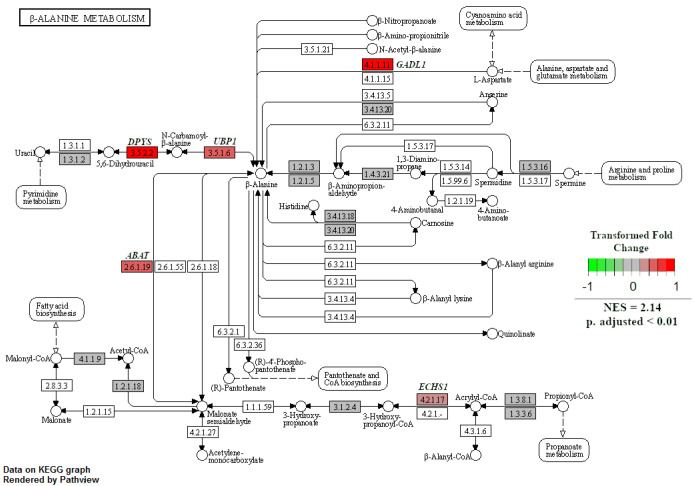
β-Alanine metabolism in NR SGA versus NR NonSGA placentomes at day 70 of pregnancy. Gene set enrichment analysis was performed to identify KEGG pathways that were significantly enriched at adjusted *p*-values < 0.05.

When GSEA analyses were performed between treatment groups to identify enriched KEGG pathways, only two pathways were significant at an adjusted *p*-value < 0.05. The first was the natural-killer-cell-mediated cytotoxicity pathway, which was enriched in NR SGA placentomes compared to 100% NRC placentomes at day 70 of pregnancy (Figure 7). Upregulated genes in this pathway included *major histocompatibility complex, class I G1* (*HLA-G1*), *intercellular adhesion molecule 1-2* (*ICAM1-2*), *UL16-binding protein 1–3* (*ULBP1-3*), *ITGAL*, *ITGB2*, *linker for activation of T cells* (*LAT*), *protein kinase C* (*PKC*), and *perforin 1* (*PRF1*), while downregulated genes included *CD94*, *Rac family small GTPase 1* (*Rac*), and *SHC adaptor protein 1* (*Shc*; Figure 7). The second enriched pathway was β-alanine metabolism in NR SGA placentomes compared to NR NonSGA placentomes at day 70 of pregnancy (Figure 8). Upregulated genes in this pathway included *dihydropyrimidinase* (*DPYS*), *upstream-binding protein 1* (*UBP1*), *glutamate decarboxylase-like 1* (*GADL1*), and *enoyl-CoA hydratase, short chain 1* (*ECHS1*), while there were no downregulated genes (Figure 8).

## 3. Discussion

Nutrient restriction during pregnancy reshapes the interaction between, and function of, the maternal, placental, and fetal compartments. Previous studies have indicated that the placenta—specifically the placentome in sheep—is not only nutrient-sensitive, but may be able to adapt to nutrient restriction as measured by the growth of the fetus [5,6]. Because placental growth precedes fetal growth, a major limitation in placental biology research—including previous studies on sheep—has been the inability to conduct a retrospective assessment of placental development responses that give rise to a spectrum of fetal growth outcomes. Using the pregnant sheep as our model organism, we developed a surgical technique to remove a single placentome during mid-gestation, and then allowed the pregnancy to proceed, which has provided the opportunity for a retrospective assessment of the early and late placentomal transcriptome based on late gestation fetal weight. Results from these analyses have revealed that a relatively low number of genes are differentially expressed between placentomes from well-fed ewes, NR ewes with NonSGA fetuses, and NR ewes with SGA fetuses—especially in comparison to the number of genes that are differentially expressed over time in the placentome—but those genes are involved in a variety of functions that lead to a broader impact later in pregnancy. Additionally, GSEA analyses have revealed a small number of unique pathways at day 70—including natural-killer-cell-mediated cell toxicity in SGA fetuses—that have interesting implications for placental development and function.

A previous study in our laboratory used a microarray analysis to identify transcriptomic changes between SGA and NonSGA placentomes from NR pregnancies at day 125 of pregnancy [6]. Major findings of that study included differentially expressed gene clusters involved in immune response, cell signaling, nutrient response, and nutrient transport [6]. These placentomes also showed histological differences, including decreased placentome volume and total maternal/fetal interface surface area in SGA pregnancies compared to NonSGA pregnancies from NR ewes [5]. The current study showed similar functional differences between SGA and NonSGA placentomes, with chemokine binding and cytokine activity altered at day 135 in addition to signaling receptor binding, nutrient transporter activity, and cell structure molecules. Indeed, certain genes–such as *CD86*, *CXCL10*, and *DPYD*—were differentially expressed in both studies, even with the small difference in timing [6]. Similar observations in late pregnancy between the two studies demonstrate that the model is reproducible, and provide confidence that DEGs identified in mid-gestation are inducing changes in placental function that lead to divergent patterns of fetal growth.

In the same cohort of animals as the current study, we previously found that triglycerides and bile acids were accumulating in allantoic fluid during mid-gestation of SGA fetuses from NR ewes, but not in allantoic fluid of NonSGA fetuses, suggesting impaired placental transport. We next found that SLC27A6 protein levels were reduced in mid-gestation placentomes from NR sheep with SGA fetuses, but not in NonSGA fetuses [9]. Interestingly, SLC27A6 is robustly expressed during placental growth, but is only modestly expressed during late gestation, highlighting a potentially novel role for this member of the fatty acid transport family in supporting early placental growth during nutritional hardship. This particular transporter family also has adaptive potential in other species, as both SLC27A2 and SLC27A6 were increased in the late-gestation baboon placenta in response to maternal nutrient restriction [14]. In addition to *SLC27A6* being differentially expressed between SGA and NonSGA placentomes from NR ewes in the current transcriptomic analysis, the molecular function of lipid kinase activity was altered, and fatty acid metabolism was noted as a pathway that was upregulated in SGA, but not NonSGA fetuses, in the GSEA analyses at day 70 of pregnancy. This connection between gene expression, protein expression, and systemic metabolic levels reinforces the value of using transcriptomic analyses to further elucidate mechanisms by which the placentome is able to adapt to nutrient restriction.

The placenta is a unique organ, in that it performs a variety of functions that would be relegated to specific organs in the adult animal. When conducting pathway analyses, this is important to consider, as the implications of certain pathways could be quite different than they would be in their intended organ. An example of this in the current study is the hallmark pathway of pancreas beta cells (M5957), which is composed of genes specifically upregulated in pancreatic beta cells. At day 70, this pathway is positively enriched in placentomes from NR pregnancies with NonSGA fetuses, but negatively enriched in NR pregnancies with SGA fetuses (Figure 5). Overall, many of the genes in the pathway that are enriched are involved in glucose and insulin regulation but, interestingly, the vitamin D receptor, *VDR*, is also enriched in this pathway in placentomes from SGA fetuses. Dysregulation of vitamin D metabolism has been implicated in intrauterine growth restriction in human pregnancies [15], and is predominantly located in the syncytial cells of the placentome during early to mid-pregnancy in sheep [16]. In addition to traditional roles in the regulation of calcium and phosphate homeostasis, vitamin D is a regulator of immune function that has been hypothesized to play an immunomodulatory role at the maternal–fetal interface [17]. Vitamin D, acting through its nuclear hormone receptor, VDR, has a large number of gene targets, and knockdown of *VDR* in mice led to increased proinflammatory cytokine and chemokine expression in placental cells, supporting a role for vitamin D in the placenta [18]. Vitamin D’s status has not been assessed in this study, but is potentially of importance for future studies.

Of interest to this study is the potential interaction of vitamin D with macrophages and natural killer (NK) cells. Vitamin D can be locally activated in the placenta by macrophages, and promotes macrophage proliferation and differentiation [19,20]. At day 70 of pregnancy, *CD163*—a macrophage-specific marker whose upregulation is associated with a response to inflammation [21]—was upregulated in NR SGA placentomes compared to 100% NRC placentomes (Table 1). Additionally, *CD163* continued to be upregulated at day 135 in NR SGA placentomes compared to both NR NonSGA and 100% NRC placentomes, indicating a potential increase in either the total population of actual macrophages, or, as the cells of the placenta tend to take on the roles of other cells, the number of cells responding to perceived inflammation to help promote tissue modeling and the scavenging of apoptotic cells, as has been seen in other uterine environments [22].

NK cells can also be regulated by vitamin D, and are used as a defense mechanism against cells undergoing forms of stress [23]. By binding ligands on a target cell’s surface, NK cells are activated to release cytotoxic granules onto the bound target cell in order to induce programmed cell death [24]. At day 70 of pregnancy, GSEA analysis showed an enrichment for the KEGG pathway natural-killer-cell-mediated cytotoxicity in placentomes from NR SGA pregnancies compared to those from well-fed pregnancies (Figure 7). At day 135, there was also upregulation of *CD96*—an NK cell marker—in SGA placentomes compared to placentomes from well-fed pregnancies and NR NonSGA pregnancies (Table 2). Multiple genes from a variety of steps in the cytotoxicity process were upregulated, from target cell ligands and NK cell receptors to *TNF-α*, genes involved in cytotoxic granule exocytosis, and apoptotic genes. Considering the histological changes, such as decreased surface area at the maternal–fetal interface in placentomes from SGA fetuses that were seen previously [5], the implications of increased cytotoxic activity may manifest in both histoarchitectural and functional aspects of placentome development. Of note, increased NK-cell-mediated cytotoxicity has also been associated with recurrent pregnancy loss in vitamin-D-deficient women, lending weight to the negative impact it can have on placental development [24,25].

Major histocompatibility complex (MHC) class II molecule expression serves as a mediator of T-lymphocyte response [26,27]. This is of interest because, at day 70, MHC class II protein binding was a molecular function that was altered in NR SGA compared to 100% NRC placentomes. MHC class II receptor activity was also the only molecular function that was altered in NR NonSGA compared to 100% NRC placentomes and, indeed, two of the three genes involved—*HLA-DQA2* and *HLA-DQB2*—were downregulated in the NR placentomes (Figure 2). While these two genes are considered to be nonfunctional pseudogenes in humans, they are transcribed and translated in sheep and cattle, although their exact roles are still unknown, and may be of interest to study in the context of placentome development [28].

One limitation to this study is that after mapping reads to the available sheep genome, those gene identifiers were then matched to human identifiers for further bioinformatics analyses, due to the availability of resources to analyze the human transcriptome as opposed to that of the sheep. This resulted in the loss of sheep-specific genes from the GSEA analyses that may have an important role that could be further elucidated by working through specific pathways of interest. The other area where this swap from sheep to human identifiers would need to be explored further is in the major histocompatibility complex molecules and NK cell complex molecules. While there are a number of similarities between the sheep and human genomes in these pathways, there are also documented differences that reinforce that these bioinformatics analyses are exploratory tools only, and specific mechanisms must be worked out directly [28,29].

Alterations in amino acid transport in the late-gestation placenta, and resultant changes in fetal plasma amino acid concentrations, have been well documented in NR pregnancies resulting in SGA offspring [5,30,31,32,33]. However, one of the pathways that were enriched in this study in SGA compared to NonSGA placentomes in mid-pregnancy that is unique is beta-alanine metabolism. Beta-alanine is a non-essential amino acid that is not incorporated into proteins, but serves as an intermediary that can be used for fatty acid synthesis, pyrimidine metabolism, or carnosine production [34]. The specific enzymes that are upregulated in the SGA fetuses appear to drive the production of beta-alanine from L-aspartate and uracil, with beta-alanine itself then being utilized in fatty acid biosynthesis (Figure 8). There appears to be little knowledge about a role for beta-alanine in the placenta, but its use could very well be an adaptive measure undertaken by the NR placentome to maintain function.

The GO term signaling receptor binding (GO:0005102) was identified as differential between SGA and NonSGA placentomes, and identifies genes that bind to a receptor molecule to initiate a change in cell function. At day 70, 9 of the 22 differentially expressed genes fit into this category, and especially interesting is that 4 of those—*FAM3C*, *MSTN*, *TNFRSF11B*, and *WNT9B*—were also identified in the cytokine activity molecular function (GO:0005125), which is defined as genes that interact with a receptor to control survival, growth, and differentiation. These genes have had little to no previous mechanistic association with placental development, especially in sheep, and have potential as critical regulators relative to their roles in other tissues. *FAM3C* has an insulin-independent regulatory role in hepatic gluconeogenesis and lipogenesis [35]. *MSTN*, a negative regulator of muscle development, may also be involved in cytokine production and glucose metabolism, and has been associated with preeclampsia and IUGR in human pregnancies [36,37]. *TNFRSF11B*, also known as osteoprotegerin, is a secreted factor that is a key regulator in bone metabolism, but is also pro-angiogenic, and has been associated with preeclampsia and diabetes mellitus in human pregnancies [38,39]. *WNT9B* drives mesenchymal–epithelial transitions in the urogenital system during organogenesis [40].

Signaling receptor binding was also identified at day 135 of pregnancy, and included 20 of the 100 differentially expressed genes between SGA and NonSGA placentomes. From the genes involved in signaling receptor binding, only *MSTN* and *MUC4* were differentially expressed at day 70 and day 135 of pregnancy. *MUC4* is a cell-surface membrane-bound glycoprotein that sterically masks cell surface antigens to protect cells from immune recognition—most notably in cancer cells [41]—and a specific role in the placenta has not been well defined. Multiple C–X–C motif chemokine family members (*CXCL8*, *CXCL9*, *CXCL10*) were upregulated in SGA placentomes, as was the interferon gamma response pathway, emphasizing that an inflammatory environment has been established in those placentomes late in pregnancy that was not necessarily active in mid-pregnancy, but was likely developing [42].

Identifying the regulators of differentially expressed genes—transcription factors—is a critical piece when trying to elucidate the mechanisms driving changes in placentome development. We chose to identify those transcription factors that may be of importance in differentiating between SGA and NonSGA placentomes during mid-gestation, because they potentially establish the developmental trajectory that the placentome will follow. Nine transcription factors were identified that were actually expressed in the NR placentomes that had potential to regulate at least 4 of the 22 DEGs from day 70, although none of them were expressed differently between SGA and NonSGA placentomes. Two of the transcription factors—*PPARG* and *SOX17*—have previously identified roles in the placenta, but the rest do not [43]. *PPARG* is essential for placental development, has functions in adipogenesis and inflammation, and has been previously shown to be nutrient-sensitive in the sheep placenta [44,45]. *GABPA* is a regulator of cellular energy metabolism and protein synthesis, as well as cytokine expression [46]. In addition to the 9 differential genes potentially regulated by *GABPA* at day 70 between SGA and NonSGA, there are 13 genes at day 135 potentially regulated by *GABPA*, including *carboxylesterase 4A* (*CES4A*), *claudin domain-containing 1* (*CLDND1*), *collagen type 25 alpha 1* (*COL25A1*), *cytohesin-4* (*CYTH4*), *G-protein-coupled receptor 182* (*GPR182*), *insulin-like growth factor-binding protein 2* (*IGFBP2*), *inosine monophosphate dehydrogenase 1* (*IMPDH1*), *ITGB2, keratin 4* (*KRT4*), *ladinin-1* (*LAD1*), and *leucine-rich repeat Ig-like transmembrane domains 1* (*LRIT1*). *GABPA* itself is stably expressed in all placentomes at both days, but interacts with a variety of cofactors, including VDR, to achieve regulation of target genes [46,47].

## 4. Materials and Methods

### 4.1. Animal Study and Tissue Collection

Mature Hampshire ewes of similar parity, frame size, and initial body condition were fed to meet 100% of their National Research Council (NRC) [48] nutritional requirements, and served as embryo transfer recipients [5,13]. Ewes were synchronized into estrus, and a single embryo from a superovulated Hampshire donor ewe of normal body condition was transferred into the uterus of a recipient ewe on day 6 post-estrus [5]. Pregnancy was diagnosed by ultrasound on day 28 of gestation. All ewes were individually housed in pens with concrete flooring from days 28 to 135 of gestation, and fed once daily. Beginning on day 28 of gestation, body weight was measured weekly, and feed intake was adjusted based on changes in body weight. On day 35 of pregnancy, ewes were assigned randomly to either a control-fed group (100% NRC; *n* = 8) or a nutrient-restricted (NR) group (50% NRC; *n* = 28); composition of their respective diets has been published previously [49]. NR ewes were provided 50% of the total weight of feed that the control-fed group received, in order to induce a total caloric restriction equally across macromolecule groups. Vitamins and minerals were provided as recommended or in excess for all ewes.

On day 70 of pregnancy, a single placentome was surgically removed, as previously described [9]. Briefly, care was taken to remove a placentome from near the antimesometrial greater curvature of the gravid uterus and proximal to the anterior end of the amniotic membrane. The placentome was finely minced and thoroughly mixed in order to ensure representation of all cell types, before being snap-frozen in liquid nitrogen for RNA analyses. Necropsies were performed on day 135 of gestation. At this time, placentomes were dissected, weighed, and then processed, as on day 70.

Fetuses from ewes fed 100% NRC were the control group (*n* = 8). Fetuses within the NR group (*n* = 28) were segregated into quartiles based on their fetal weights. The highest (NR NonSGA; *n* = 7) and lowest (NR SGA; *n* = 7) quartiles were selected for further investigation [10,12].

### 4.2. RNA Extraction, Sequencing, and Analyses

Total RNA was extracted from snap-frozen placentomes using TRIzol reagent (Invitrogen, Carlsbad, CA, USA), according to the manufacturer’s recommendations. Extracted RNA was treated with DNase I (Qiagen, Hilden, Germany) and purified using a RNeasy Mini Kit (Qiagen), before the RNA was quantified and its quality was assessed using a NanoDrop and an Agilent 2100 Bioanalyzer (Agilent Technologies, Santa Clara, CA, USA), respectively. An RNA integrity number (RIN) of >8 and a 260/230 value of >1.8 were considered acceptable. Extracted RNA was stored at −80 °C until further analyses.

Total RNA from the samples was submitted to the University of Missouri DNA Core facility (Columbia, Missouri, USA). Library construction and sequencing was conducted following the manufacturer’s protocol with reagents supplied in Illumina’s TruSeq Stranded mRNA sample preparation kit. Libraries were multiplexed and sequenced from both directions as 75 base pair paired-end reads on one lane on a NextSeq500 instrument. The raw sequences (FASTQ) were subjected to quality check via FastQC (http://www.bioinformatics.babraham.ac.uk/projects/fastqc/ (accessed on 16 July 2017)). The program fqtrim (https://ccb.jhu.edu/software/fqtrim/ (accessed on 16 July 2017)) was used to remove adapters, perform quality trimming (phred score > 30) by a sliding window scan (6 nucleotides), and select read lengths of 30 nucleotides or longer after trimming. The reads obtained from the quality control step were mapped to the sheep reference genome (Oar_v3.1) using Hisat2 aligner [50]. The program FeatureCounts [51] was used to quantify read counts by using the sequence alignment files of each sample. Genes with evidence of expression (counts per million; rowSum > 5) were used for model-based differential expression analysis using the edgeR robust method [52]. The false discovery rate (FDR) ≤ 0.10 was used as threshold for statistically significant differential expression of genes. Only protein-coding genes were included. Venn diagrams were produced using Venny 2.1 (http://bioinfogp.cnb.csic.es/tools/venny/index.html (accessed on 13 May 2021)). Over-representation analyses using DEG lists were conducted using ToppFun (http://toppgene.cchmc.org/ accessed on 18 May 2021)) with default settings [53] to identify Gene Ontology (GO) terms for the molecular function ontology (FDR < 0.05). Sheep Ensembl IDs were converted to human Ensembl IDs (genome assembly GRCh38.p13) in order to facilitate pathway analyses. Enrichment analyses were performed using gene set enrichment analysis software v4.1.0 (http://gsea-msigdb.org/ (accessed on 14 May 2021)) [54], with statistical significance set at an FDR *q*-value < 0.25. Transcription factors were identified using the Tf2DNA database (http://fiserlab.org/tf2dna_db/ (accessed on 18 May 2021)). KEGG pathway analyses were performed using the pathview package in R [55]. Data files were deposited in the National Center for Biotechnology Information (NCBI) Gene Expression Omnibus (GEO) under accession number GSE180182.

## 5. Conclusions

The results of the present study retrospectively identify novel DEGs and pathways during mid-gestation that give rise to a spectrum of fetal weight phenotypes in late gestation. Maternal nutrient restriction appears to trigger alterations in lipid metabolism, leading to a proinflammatory state that initiates a cascade of immune effects that are maintained into late gestation, specifically in those placentomes from pregnancies that produce SGA fetuses. In contrast, the placentomes from pregnancies with NonSGA fetuses are able to adapt to nutritional hardship, as evidenced by transcriptome changes in mid-pregnancy, in order to avoid the fate of the placentomes from the SGA fetuses, and become similar to the placentomes from well-fed control pregnancies in late gestation. Future studies are necessary to investigate the influence of identified key genes, such as *MSTN*, and potential systemic effectors, such as vitamin D, in order to be able to devise potential therapeutics to alleviate pregnancies resulting in small-for-gestational-age offspring.

## Figures and Tables

**Table 1:** Differentially expressed genes in placentomes at day 70 of pregnancy.

NR SGA ^1^ vs. 100% NRC			NR NonSGA vs. 100% NRC			NR SGA vs. NR NonSGA		
Gene	Log2 FC ^2^	FDR ^3^	Gene	Log2 FC	FDR	Gene	Log2 FC	FDR
*MSTN*	−3.10	0.03	*HNRNPK*	−6.28	0.01	*HNRNPK*	−5.53	0.02
*CSTF2T*	−2.78	0.08	*UBE2D3*	−3.36	<0.01	*MSTN*	−3.74	0.03
*HLA-DQA1*	−1.98	0.03	*CSTF2T*	−3.17	0.02	*FEM1A*	−2.35	0.09
*SLC15A5*	−1.92	0.07	*GBP4*	−2.74	0.01	*EEF1A1*	−1.91	0.06
*COL19A1*	−1.87	0.01	*PPAT*	−2.23	0.09	*AMER3*	−1.56	0.10
*IL12RB2*	−1.52	0.01	*OLFM1*	−1.81	0.09	*HLA-DOB*	−1.28	0.07
*GPRIN2*	−1.12	0.02	*APLP1*	−1.44	0.01	*MUC4*	−1.14	0.07
*CD74*	−1.01	<0.01	*HLA-DQB2*	−1.38	0.09	*SLC27A6*	−0.77	0.06
*FZD2*	−0.85	0.05	*HLA-DQA2*	−1.04	0.01	*WNT9B*	−0.76	0.05
*MMP9*	−0.75	0.10	*NOTUM*	−0.61	0.10	*JAM2*	−0.73	0.08
*PI15*	−0.73	<0.01	*SH3GL3*	−0.44	0.01	*APOE*	−0.67	0.06
*LRATD1*	−0.50	0.08	*MCM5*	−0.35	0.05	*PAM*	−0.54	0.02
*NFE2L3*	−0.35	0.08	*GINS2*	0.34	0.09	*FAM3C*	0.35	0.06
*THBS1*	0.58	0.10	*TTC21B*	0.43	0.10	*ZNF462*	0.53	0.04
*CD163*	0.65	0.06	*PAM*	0.50	0.01	*AQP3*	0.87	0.06
*NDUFA4L2*	0.76	0.08	*JMY*	0.50	0.09	*S100B*	1.04	0.02
*PRSS12*	0.82	0.01	*MRC1*	0.51	0.03	*MYO1A*	1.28	0.06
*PLAC8L1*	0.91	0.03	*RNASE1*	0.57	0.09	*DOK5*	1.31	0.07
*CACNG3*	1.78	0.08	*FOLR3*	0.60	0.01	*EVA1A*	1.32	<0.01
*BTNL9*	1.92	0.07	*FHL1*	0.66	0.01	*TNFRSF11B*	2.07	0.06
*SPATS1*	2.31	0.08	*SGCB*	0.67	0.03	*GFRA2*	2.28	0.02
*SLC5A12*	2.53	0.08	*NIPAL4*	0.78	0.09	*SLC10A6*	2.77	0.06
			*ZC3HAV1L*	0.80	0.09			
			*SVOPL*	0.83	0.01			
			*HLA-DOB*	1.35	0.05			
			*RPL6*	1.46	0.01			
			*PDE6B*	2.13	0.01			

^1^ 100% NRC: well-fed controls; NR NonSGA: nutrient-restricted normal-weight fetuses; NR SGA: nutrient-restricted low-weight fetuses. ^2^ Log2 FC: log2 fold change. ^3^ FDR: false discovery rate.

**Table 2:** Differentially expressed genes in placentomes at day 135 of pregnancy.

NR SGA ^1^ vs. 100% NRC			NR NonSGA vs. 100% NRC			NR SGA vs. NR NonSGA Cont.		
Gene	Log2 FC ^2^	FDR ^3^	Gene	Log2 FC	FDR	Gene	Log2 FC	FDR
*CLRN2*	−3.82	0.04	*CYP3A5*	−3.68	0.04	*LPCAT4*	−0.48	0.02
*PIWIL1*	−3.34	0.09	*CYP3A24*	−3.36	0.03	*GSTA4*	−0.45	0.08
*MSTN*	−3.19	0.10	*ARSD*	−1.43	0.04	*IMPDH1*	−0.45	0.10
*NTNG1*	−2.85	0.06	*GLRX5*	1.08	0.04	*ATL1*	−0.44	0.08
*CCL11*	−2.51	0.09	*RPL6*	1.59	<0.01	*ADAM15*	−0.42	0.10
*CCDC70*	−2.16	0.07	*DDX4*	1.72	0.07	*DGKZ*	−0.40	0.06
*SEMA3E*	−2.06	0.08	*H2AC18*	5.33	0.03	*LAPTM5*	0.37	0.04
*PPIL6*	−1.65	0.05	NR SGA vs. NR NonSGA			*ZNFX1*	0.39	0.09
*CLIC6*	−1.63	0.02				*CLEC1A*	0.45	0.03
*CAPN11*	−1.50	0.10	Gene	Log2 FC	FDR	*SLC38A7*	0.47	0.10
*ANPEP*	−1.17	0.03	*MSTN*	−4.25	<0.01	*OLA1*	0.49	0.04
*IL1R2*	−1.11	0.08	*KRT4*	−3.76	0.06	*CTSK*	0.57	0.01
*CASQ1*	−0.99	0.01	*CLRN2*	−3.49	0.08	*TLR2*	0.60	0.02
*SH3BP2*	−0.79	0.08	*MAN2B2*	−2.73	<0.01	*KCNJ8*	0.61	0.10
*CEP295NL*	−0.78	0.02	*ANGPTL7*	−2.20	0.04	*SERPINB2*	0.70	0.10
*ETNPPL*	−0.69	0.02	*COL25A1*	−2.08	0.10	*IFI44L*	0.72	0.09
*CCDC80*	−0.67	0.02	*CCDC151*	−1.97	0.01	*ITGB2*	0.75	0.04
*HAPLN3*	−0.64	0.10	*RPL6*	−1.88	<0.01	*GALNT13*	0.78	0.03
*ATL1*	−0.51	0.04	*LRIT1*	−1.80	0.09	*DPYD*	0.79	0.10
*CHRNE*	−0.43	0.10	*OSTN*	−1.79	0.02	*CYTH4*	0.79	0.05
*CTSK*	0.54	0.01	*MUC4*	−1.75	0.07	*CD274*	0.87	0.10
*FCGR1B*	0.73	0.09	*TCL1B*	−1.74	<0.01	*CMTM4*	0.89	0.10
*UMAD1*	0.75	0.06	*CCDC86*	−1.63	0.09	*CLEC4A*	0.92	0.01
*CLEC4A*	0.79	0.10	*MYOM3*	−1.33	0.08	*IGFBP2*	0.96	0.05
*MAF*	0.83	0.09	*PCYOX1*	−1.25	0.01	*CLEC4G*	0.98	0.03
*COLGALT2*	0.98	0.01	*ERICH4*	−1.21	0.10	*CD163*	0.99	<0.01
*FST*	1.01	0.08	*RPL30*	−1.14	0.06	*LRRC25*	1.02	0.09
*CD163*	1.02	0.01	*PODN*	−1.11	0.01	*ITGB3*	1.05	0.05
*CD200R1L*	1.03	0.06	*CES4A*	−1.04	0.01	*EPHB1*	1.09	0.10
*NRN1*	1.09	0.10	*CASQ1*	−1.02	0.02	*GPR182*	1.11	0.09
*CLEC4G*	1.11	0.02	*RLN3*	−1.00	0.09	*ITGAM*	1.15	0.01
*LRRC31*	1.14	0.02	*CCNA1*	−0.94	0.05	*SLAMF6*	1.19	0.08
*ISG15*	1.20	0.10	*HSH2D*	−0.94	0.07	*EVA1A*	1.26	<0.01
*IFIT1*	1.21	0.01	*CEP295NL*	−0.90	<0.01	*CD86*	1.30	<0.01
*SLAMF6*	1.21	0.02	*FIG4*	−0.90	0.04	*LAD1*	1.37	0.10
*EPM2A*	1.29	0.06	*TST*	−0.89	0.08	*ISG15*	1.47	0.04
*CD96*	1.37	0.07	*CYP8B1*	−0.89	0.06	*CXCL8*	1.47	<0.01
*ERVW-1*	1.44	0.06	*SLC13A3*	−0.82	<0.01	*CXCL9*	1.57	0.04
*P2RX2*	1.45	0.04	*CLCN1*	−0.77	0.05	*CD2*	1.57	0.08
*TRIML2*	1.54	0.01	*A2ML1*	−0.75	0.02	*IFIT1*	1.59	<0.01
*SH2D2A*	2.01	0.07	*ADA*	−0.66	0.06	*BMP3*	1.69	0.08
*CXCL10*	2.25	0.10	*ORAI2*	−0.65	0.09	*CLGN*	2.06	0.09
*ZNF623*	3.25	0.01	*CRYAB*	−0.64	0.05	*CXCL10*	2.11	0.03
*SNX10*	3.47	0.07	*NINL*	−0.63	0.06	*CLEC4F*	2.17	0.09
*CSTF2T*	3.65	<0.01	*AGK*	−0.61	0.10	*CD96*	2.49	<0.01
*RPL9*	4.37	0.05	*CALML5*	−0.61	0.03	*SAXO1*	3.31	0.08
*H2AC18*	5.56	<0.01	*CLDND1*	−0.60	0.01	*CSTF2T*	3.36	<0.01
			*ART4*	−0.58	0.10	*CYP3A5*	3.40	0.01
			*OTULINL*	−0.55	0.04	*RPL9*	4.36	0.06
			*CRELD2*	−0.53	0.04	*CCDC148*	4.52	<0.01
			*FBLN1*	−0.51	0.04			

^1^ 100% NRC: well-fed controls; NR NonSGA: nutrient-restricted normal-weight fetuses; NR SGA: nutrient-restricted low-weight fetuses. ^2^ Log2 FC: log2 fold change. ^3^ FDR: false discovery rate.

## Data Availability

Data files were deposited in the National Center for Biotechnology Information (NCBI) Gene Expression Omnibus (GEO) under accession number GSE180182.
